# CD8α and CD70 mark human natural killer cell populations which differ in cytotoxicity

**DOI:** 10.3389/fimmu.2025.1526379

**Published:** 2025-02-19

**Authors:** Camille Rey, Katherine L. Jones, Kevin B. Stacey, Alicia Evans, Jonathan D. Worboys, Gareth Howell, Sam Sheppard, Daniel M. Davis

**Affiliations:** ^1^ Faculty of Biology Medicine and Health, Lydia Becker Institute of Immunology and Inflammation, Manchester, United Kingdom; ^2^ Department of Life Sciences, Sir Alexander Fleming Building, Imperial College London, South Kensington, London, United Kingdom

**Keywords:** NK cells, CD8, CD70, cytotoxicity, RNA sequencing, cancer immunotherapy, serial killing, NK cell therapy

## Abstract

Natural Killer (NK) cells are innate immune cells that can directly detect and kill cancer cells. Understanding the molecular determinants regulating human NK cell cytotoxicity could help harness these cells for cancer therapies. To this end, we compared the transcriptome of NK cell clones derived from human peripheral blood, which were strongly or weakly cytotoxic against 721.221 and other target cells. After one month of culture, potent NK cell clones showed a significant upregulation in genes involved in cell cycle progression, suggesting that proliferating NK cells were particularly cytotoxic. Beyond two months of culture, NK cell clones which were strongly cytotoxic varied in their expression of 28 genes, including *CD8Α* and *CD70*; NK cells with high levels of *CD70* expression were weakly cytotoxic while high *CD8Α* correlated with strong cytotoxicity. Thus, NK cells were cultured and sorted for expression of CD70 and CD8α, and in accordance with the transcriptomic data, CD70^+^ NK cells showed low cytotoxicity against 721.221 and K562 target cells. Cytotoxicity of CD70^+^ NK cells could be enhanced using blocking antibodies against CD70, indicating a direct role for CD70 in mediating low cytotoxicity. Furthermore, time-lapse microscopy of NK cell-target cell interactions revealed that CD8α^+^ NK cells have an increased propensity to sequentially engage and kill multiple target cells. Thus, these two markers relate to NK cell populations which are capable of potent killing (CD70^-^) or serial killing (CD8α^+^).

## Introduction

NK cell recognition of cancer cells, virus-infected cells and other targets, is facilitated via a wide array of germline-encoded activating and inhibitory receptors (1–3). As a result, NK cells are extremely diverse in their phenotype and function (4–8). *In vitro* analyses have underscored this heterogeneity at the functional level, with observations that a single NK cell may eliminate up to ten cancer cells consecutively, whilst others may only kill one or none (4, 9, 10). Efforts have focused on understanding how specific receptor/ligand interactions regulate NK cell cytotoxicity (11). However, it is likely that many intracellular proteins also regulate NK cell cytotoxicity. One example identified so far is that NK cell education or licensing can reduce phosphatase localization at immune synapses to boost NK cell-killing potential (12).

NK cells are an attractive alternative to T cells for immune therapies, as they are capable of identifying and killing transformed cells without prior sensitization to specific target-associated antigens. Moreover, there is evidence that in allogeneic settings, NK cells have a reduced risk of eliciting graft-versus-host disease (13, 14). However, NK cells represent a small fraction of circulating peripheral blood mononuclear cells (PBMCs) and have a relatively short lifespan (15). To obtain sufficient quantities of NK cells with sustained cytolytic potential for adoptive therapy, peripheral blood NK cells must be expanded and activated *ex vivo* using feeder cells and cytokines, such as interleukin-2 (IL-2) (16, 17). Therefore, as well as improving our basic understanding of NK cell biology, identifying the cytolytic determinants of expanded NK cells may help identify populations of NK cells that are particularly useful clinically.

NK cell phenotypes change upon activation and differentiation in a context-dependent manner, leading to vast intra- and inter-individual variability of the NK cell repertoire and its functionality (18–20). Several Cancer Genome Atlas genome studies have linked NK cells with survival in some cancers (21). Based on this, investigating NK cells derived from healthy and long-term cancer-remittent donors may provide insight into NK determinants of the anti-cancer response.

Thus, we sought to investigate the intrinsic properties that drive NK cell cytotoxicity against target cells, and to identify surface proteins that could be used to define the most potently cytotoxic NK cells. To this end, NK cell clones were derived from the peripheral blood of cancer-remittent and healthy donors and assessed their cytotoxicity against 721.221 and K562 cancer target cells. Potent and weakly cytotoxic NK clones were analyzed by RNA-sequencing at different time points in culture. At 4 weeks, potently cytolytic NK clones overexpressed genes and pathways involved in cellular proliferation. At later culture time points, expression of *CD8Α* and a lack of *CD70* expression were associated with elevated cytotoxicity. Sorting NK cells based on the expression of the human glycoproteins encoded by CD8α and CD70 validated that the CD8α^+^CD70^-^ NK cell subset is particularly cytotoxic. CD70 expression increased upon NK cell culture in IL-2, and CD70 blockade enhanced cytotoxicity. CD8α expression was more stable and correlated with the ability of NK cells to sequentially kill cancer targets.

## Results

### RNA sequencing of NK clones reveals a correlation between overexpression of cellular proliferation genes and potent cytotoxicity

**Figure 1:**
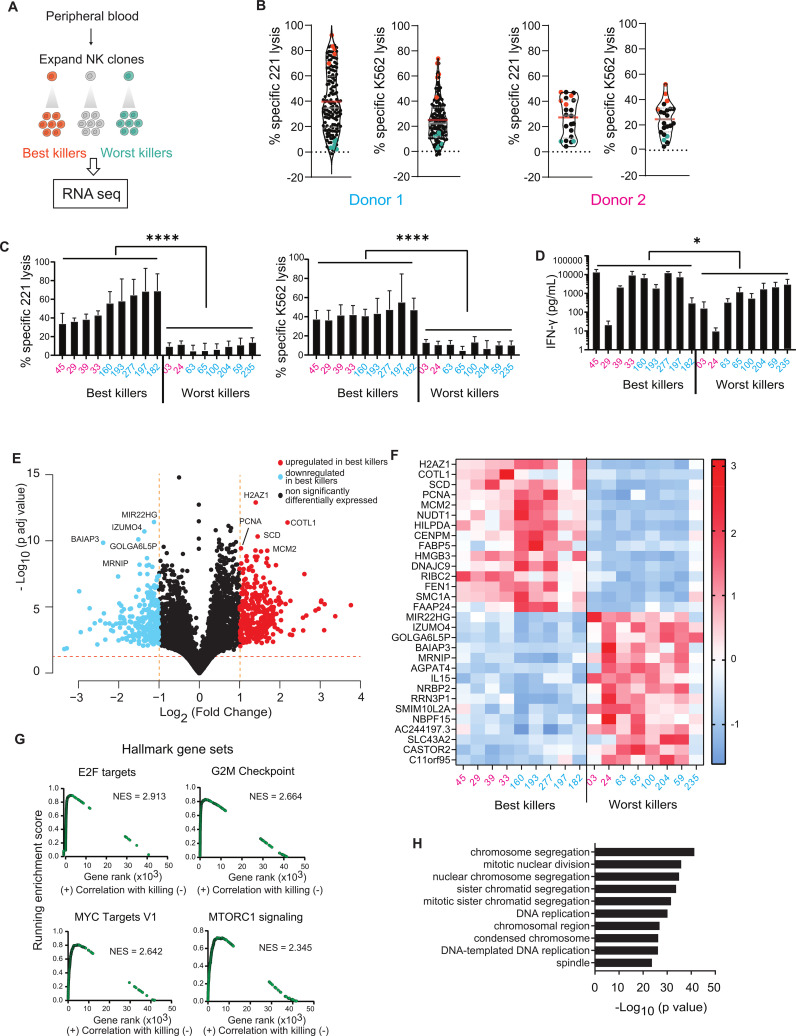
An RNA screen of short-term cultured NK clones links cellular proliferation gene expression to cytotoxicity. **(A)** Scheme of the strategy used to investigate what genes make an NK cell better or worst at killing cancer targets. **(B)** Violin plots representing Annexin-V/PI cytotoxicity profiles of NK clones derived from donors 1 and 2 against 721.221 and K562 targets, at the time point of RNA extraction. Best killer NK clones (orange dots, selected for RNA sequencing), intermediary killer NK clones (black dots) and worst killer clones (teal dots, selected for RNA sequencing) are represented. Mean and median cytotoxicity levels are represented by red and grey lines, respectively. 0.5:1 E:T ratio, 4-hour assay. **(C)** Cytotoxicity levels of selected best and worst killer NK clones across donors against 721.221 and K562 cancer targets. Individual clones are identified by numbers, donor 1 in cyan and donor 2 in pink. **(D)** IFN-γ release of selected best and worst NK clones, upon stimulation via MICA. **(C, D)** Data from three independent experiments. Mean and ± SD are *indicated.* *P<0.05; ****P<0.0001 calculated by unpaired t-tests. **(E)** Volcano plot of the differentially expressed genes *(*Log_2_ fold change ≤ -1 or ≥ 1 and a p value below 0.1) between best and worst killer NK clones. Genes significantly upregulated (red dots) and downregulated (blue dots) in the best killer NK cells are depicted alongside non-significant genes (black dots). The top 10 differentially expressed genes are labelled. **(F)** Heatmap of z-transformed normalized expression levels of top 30 differentially expressed genes, across best and worst killer clones. Relative gene expression levels are color-coded using a scale based on Z=score distribution, with the largest value (3.1044) in red, lowest value (-1.61367) in blue, and the baseline value (0) in white. **(G)** Top 4 hallmark gene sets enriched in best killer NK clones determined by comparing Normalized enrichment scores (NES) generated using GSEA. **(H)** Top 10 biological processes and chemical components significantly upregulated in best killer NK clones.

To seek determinants of NK cell cytotoxicity, we compared the transcriptome of cloned NK cells according to their levels of cytotoxicity (**Figure 1A**). In the first instance, we derived NK cell clones from two cancer-remittent patients, donors 1 and 2 (**Figure 1B**). Peripheral blood mononuclear cells (PBMCs) were isolated, and single CD56^+^CD3^-^ NK cells were sorted and expanded on feeder cells in the presence of phytohemagglutinin (PHA) and IL-2. Early in clonal expansion, after 4 to 5 weeks of culture, NK cell clones were functionally assessed for cytotoxicity and cytokine release. In parallel, total RNA was extracted from individual clones and stored for analysis.

NK clone cytotoxicity against 721.221 and K562 target cells was monitored by flow cytometry, using Annexin-V/PI to stain dead cells at an effector-to-target (E:T) ratio of 0.5:1. NK clones showed different levels of cytotoxicity (**Figure 1B**), in accordance with previous work (22). On the day of RNA extraction, the ‘best’ killing NK clones from donors 1 and 2 lysed 81 ± 8% and 42 ± 4% 721.221 target cells and 61 ± 12% and 42 ± 8% K562 target cells, respectively. In contrast, the ‘worst’ NK cell clones lysed 5 ± 3% and 8 ± 0% 721.221 target cells and 9 ± 5% and 10 ± 2% K562 target cells, respectively (**Figure 1B**, colored data points). The ‘best’ and ‘worst’ killing clones selected for further analysis were those which fulfilled several criteria: they were consistently strongly or weakly cytotoxic against both tested target cell lines, they released interferon-gamma (IFN-γ) in response to stimulation with the NKG2D-ligand MICA, and we could obtain sufficient numbers of cells for repeated assays and RNA extraction. Clones that were intermediate in their killing ability, inconsistently cytotoxic between target cell lines or did not sustain necessary cell numbers over time for RNA extraction were discarded (**Figure 1B**, black data points). Repeated assays validated significant differences in cytotoxicity between potent and weakly cytotoxic killer NK clones (**Figure 1C**). Weakly killing NK clones were able to produce a cytokine response upon stimulation with glass slides coated with MICA (**Figure 1D**). Thus, the two sets of clones were not simply ‘responsive’ and ‘unresponsive’ but rather showed specific variation in their responsiveness. Potent killers did release more IFN-γ on average, but there was wide variation in levels of cytokine secretion across both sets of NK cell clones (**Figure 1D**).

To elucidate the transcriptional signatures which may underly the observed differences in cytotoxicity, RNA was isolated on days 29 to 34 post-culture, and sequenced, for nine potent clones (five and four from donors 1 and 2, respectively) and eight weakly cytotoxic clones (six and two from donors 1 and 2, respectively). Differential gene expression analysis uncovered the expression of 569 genes, which significantly differed between the ‘best’ and ‘worst’ killing clones, with a 2-fold change and a p adjusted-value below 0.1 (**Figures 1E, F**). Amongst genes significantly upregulated in good killers, *CD38*, a gene encoding an ectoenzyme involved in transmembrane signaling, was identified. CD38 has been previously linked with NK cell cytotoxicity (23). A number of other membrane-bound proteins, which have not yet been extensively studied, such as *IFITM2, IFITM3, SLC4A10* and *TMEM97* were also identified, the latter targetable by various agents and implicated in Alzheimer’s (24, 25). Other upregulated genes such as *COTL1*, *WDR34* and *TUBB* are involved in cytoskeletal regulation and have either been directly or indirectly shown to regulate immune synapse formation in other cell types (26, 27). Numerous genes, including *FADS3*, *LMBR1L, SPRY2* and *TMEM150A*, found to be upregulated in weakly cytotoxic NK clones, could potentially play a role in dysregulating the organization of lipid rafts and signaling at the immune synapse (28).

To assess whether sets of differentially expressed genes could help us understand the differences between good and bad killer NK clones, a gene set enrichment analysis (GSEA) was performed (29). GSEA revealed the significant enrichment of hallmark cell-proliferation gene sets (E2F Targets, G2M checkpoint, MYC Targets version 1 and MTORC1 signaling) in the most potent clones (**Figure 1G**, **Supplementary Table 2**). Moreover, we identified the top biological processes and chemical components distinguishing good and bad killer clone transcriptomes using a gene ontology (GO) analysis (29). GO analysis revealed the significant upregulation of biological pathways that regulate the cell cycle and cellular division (**Figure 1H**, **Supplementary Table 3**). In contrast, genes overexpressed in weakly cytotoxic clones did not show enrichment for any hallmark gene sets or specific biological processes.

Taken together, we observed that after 4 to 5 weeks of culture, differentially expressed genes linked to enhanced NK clone cytotoxicity are mostly involved in cellular proliferation. Few of the genes identified encoded cellular surface proteins that can be easily targetable. We therefore sought to examine whether later time points, beyond the initial period of increased cellular proliferation in culture, NK clone transcriptomes of good and bad killers would reveal other types of proteins which may be involved in regulating cytotoxicity.

### RNA-sequencing of longer-term cultured NK clones reveals an association between expression of *CD8Α* and *CD70* with cytotoxicity

**Figure 2:**
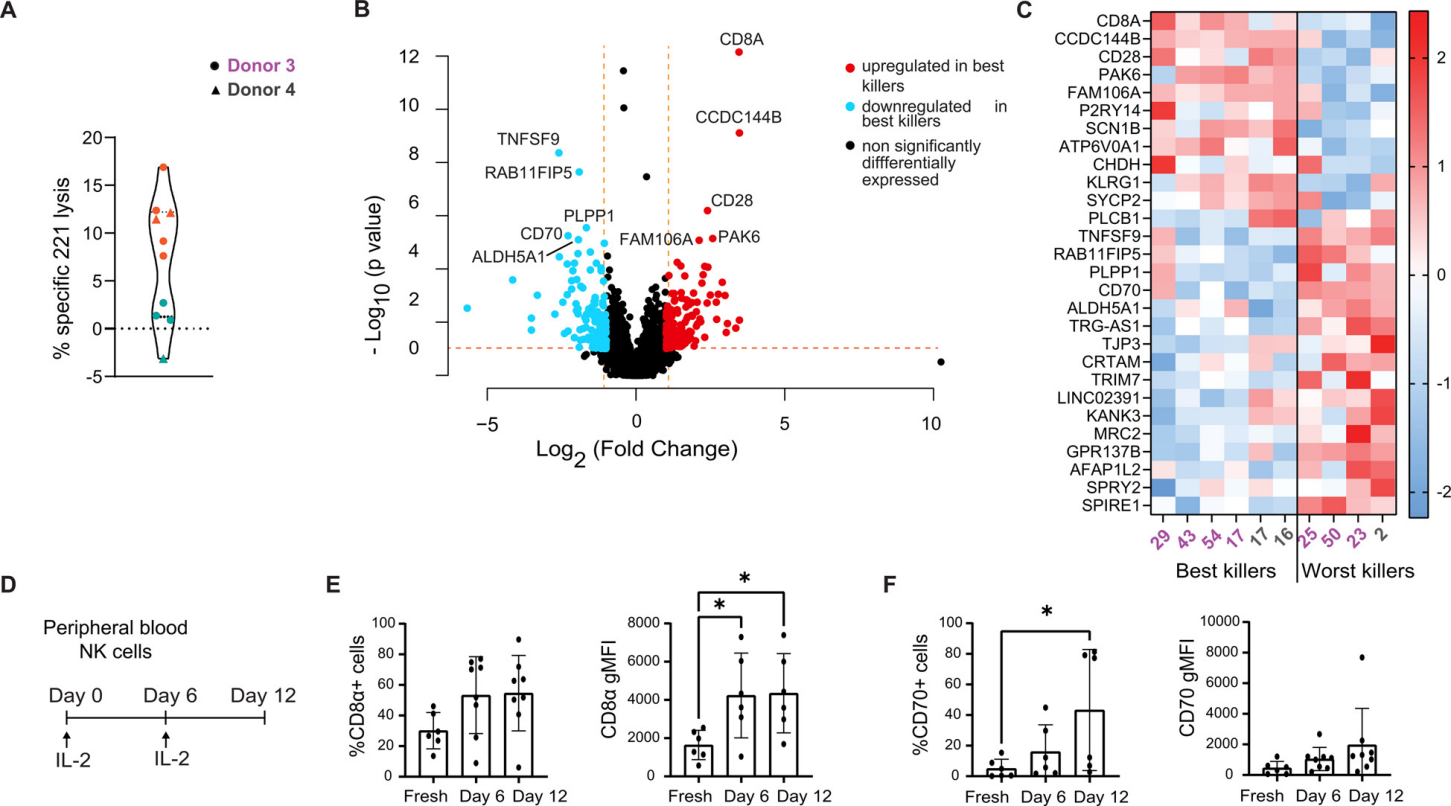
*RNA-sequencing of NK clones cultured long-term correlates CD8Α* and *CD70* expression with cytotoxicity. **(A)** Violin plot representing the cytotoxicity levels of NK clones cultured long-term, derived from donors 3 and 4, at the time point of RNA extraction. Donor 3 and 4 clones are represented with circles and triangles, respectively. Best and worst killer NK clones are represented in orange and teal, respectively. **(B)** Volcano plot of the differentially expressed genes (Log_2_ fold change ≤ -1 or ≥ 1 and a p value below 0.1) between best and worst killers among NK clones cultured long-term, across donors 3 and 4. Genes significantly upregulated (red dots) and downregulated (blue dots) in the best killer NK cells are depicted alongside non-significant genes (black dots). The top 10 differentially expressed genes are labelled. **(C)** Heatmap of z-transformed normalized expression levels of 28 differentially expressed genes, across selected best/worst killer NK clones, cultured long-term. Relative gene expression levels are colour-coded using a scale based on Z-score distribution, with the highest value (2.43205) in red, lowest value in blue (-2.23575), and baseline value (0) in white. Individual clones are identified by numbers, donor 3 in violet, donor 4 in grey. **(D)** Schematic of the extended culture protocol of freshly isolated peripheral NK cells. **(E)** Time course of percentage positive CD8α cells above isotype control and geometric mean intensity. **(F)** Time course of percentage positive CD70 cells above isotype control and CD70 geometric mean intensity. **(E, F)** Data from 6 individual donors. Mean and ± SD are indicated. *P<0.05, **(E)** calculated by one-way ANOVA, **(F)** calculated by non-parametric one-way ANOVA (Friedman test).

To explore the impact of long-term culture on the regulation of NK cytotoxicity, we isolated and expanded NK cell clones derived from peripheral blood of a long-term cancer-remittent patient (donor 3) and a healthy volunteer (donor 4) over an extended period of 2-5 months, prior to assessment of cytotoxicity by a standard radioactive release assay (**Figure 2A**). Clones derived from donors 3 and 4 were cultured for 2-5 months and 2 months, respectively.

As established in previous studies (30), it was observed that cytotoxicity declined over time and many NK cell clones ceased to proliferate over this culture period (and were thus eliminated from further analysis). We performed RNA sequencing using the six most potent and four most weakly cytotoxic NK cell clones. At the time of RNA extraction, on days 58 to 135 post-culture, the ‘best’ killing clones lysed a relatively low fraction of target cells (8-17%), whilst poor killers showed negligible cytotoxicity. Differential gene expression analysis revealed that the expression of 28 genes significantly differed between these ‘best’ and ‘worst’ killing clones, with a 2-fold change and a p adjusted-value below 0.1. Surprisingly, no obvious overlap was noted between significant genes distinguishing good and bad killer NK clones cultured long-term versus clones cultured short-term. (**Figures 2B, C**). Interestingly, *bona fide* activating receptors such as natural cytotoxicity receptors, NKG2D or DNAM-1, were not differentially regulated (2). However, an enrichment for *SIGLEC7* (31) and *CD28* (32) was observed in potent killers, both of which have known involvement in cytotoxicity. Various genes, which have not previously been established as being important for NK cell cytotoxicity were also differentially expressed, such as one encoding for an immune checkpoint on T cells, *KLRG1* (33, 34), and the *PAK6* kinase (35). Weakly cytotoxic clones overexpressed *RAB11FIP5*, a marker of NK cell dysregulation in HIV-1 patients (36); and *CD80*, encoding a CD28 ligand [**Figures 2B, C** (32, 37)]. Thus, a distinct transcriptional signature relating to cytotoxicity was identified in NK clones which persisted in culture. *CD8Α* and *CD70* were identified as top hits encoding for transmembrane glycoproteins in the most potent and weak killers, respectively. Thus, *CD8A* and *CD70* were selected for further investigation.

### Isolation and phenotyping of NK cell subsets

To further test the role of *CD8Α* and *CD70*, we first analyzed the expression of these markers at the protein level using flow cytometry. The proportion of cells positive for these proteins and their expression level were low in freshly isolated peripheral blood NK cells. However, this increased over time in culture with IL-2 (**Figures 2D–F**). On average, the CD8α^+^ NK cell population increased to 50% after 6 days of culture with IL-2. Meanwhile, the CD70^+^ NK cell population increased from 5% to 34% after 12 days of culture with IL-2. As well as more cells showing clear expression of these proteins, the level at which they were expressed at the NK cell surface also increased upon culture in IL2.

**Figure 3:**
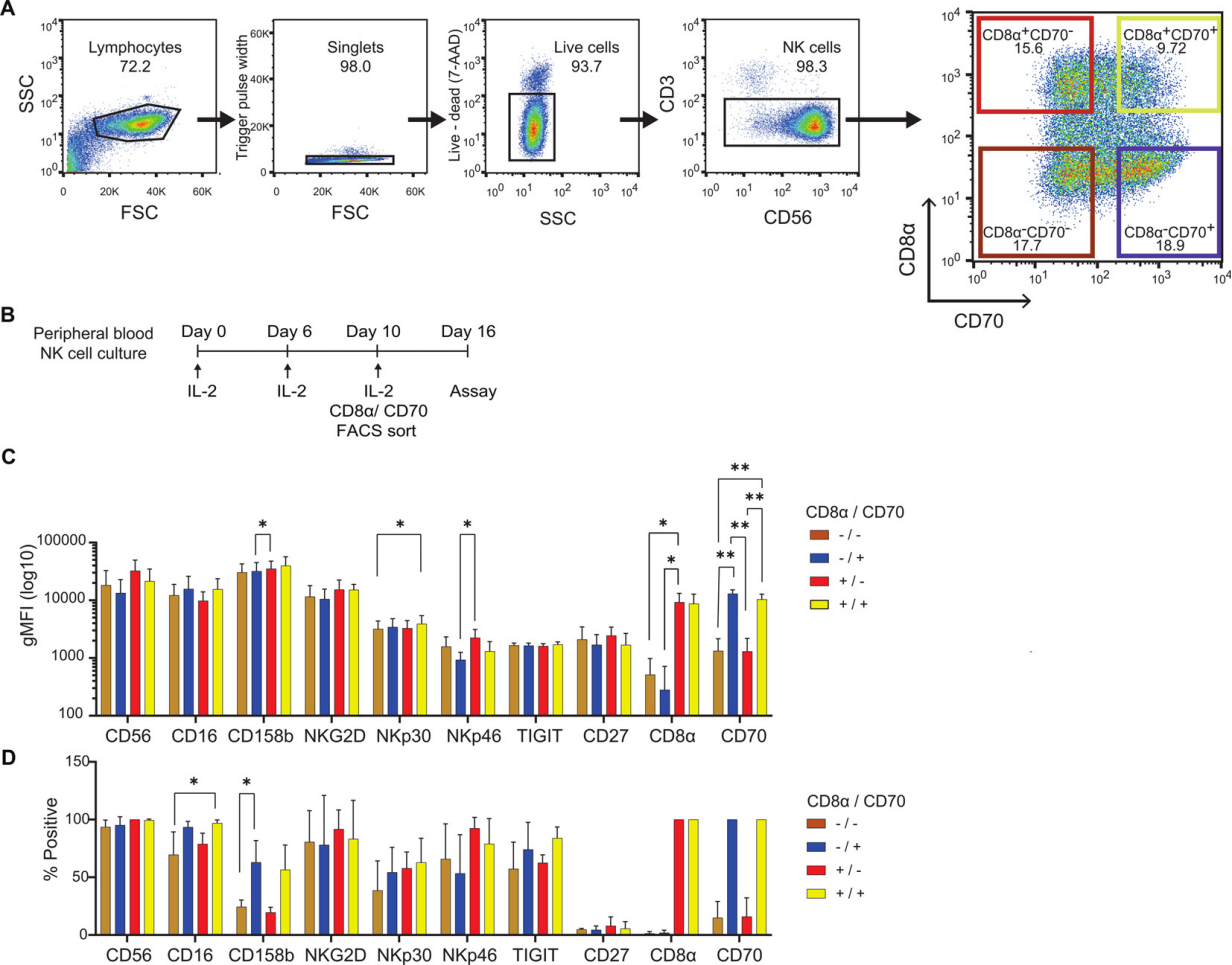
Extended culture, sorting strategy and phenotypes of CD8α/CD70 NK cell subsets. **(A)** Sorting strategy of day 10 rested live CD56+CD3- NK cells, based on CD8α and CD70 expression. Representative data. **(B)** Timeline of the extended culture and CD8α/CD70 sorting procedure of peripheral NK cells prior to functional assays and phenotyping. **(C)** Geometric mean intensity of markers of interest in different CD8α/CD70 subsets for cells showing expression above the fluorescence minus one (FMO) control. **(D)** Percentage positive cells for markers of interest in the different CD8α/CD70 subsets, above FMO control. **(C, D)** Data from 4 individual donors. Mean and ± SD are indicated. *P<0.05; **P<0.01 calculated by one-way ANOVA for individual markers.

To establish whether the expression of CD8α or CD70 correlates with known markers of cytotoxicity, NK cells were isolated by fluorescence-activated cell sorting (FACS) according to their expression of CD8α and CD70 at 10 days post IL-2 expansion (**Figures 3A, B**). The phenotype of NK cell subsets was then analyzed 6 days post-sorting using a 12-colour panel of antibodies that target a variety of NK activating (CD16, NKG2D, NKp30, Nkp46), inhibitory (CD158b, TIGIT) and maturation markers (CD27) (**Figures 3C, D**, **Supplementary Table 1**, **Supplementary Figure 2**). Across the subsets, no significant differences were noted in proportions of positive cells or geometric mean fluorescence intensities (gMFI) for CD56 (94% to 100% of the cells were CD56^bright^ across subsets), NKG2D (present on 78 to 92% of cells across subsets) or CD27 (the receptor for CD70 and a marker of immature NK cells, of which the cells were largely devoid of expression; between 4 and 6% were CD27^+^ across subsets). However, the presence of higher proportions of CD16^+^ (a receptor mediating antibody-dependent cellular cytotoxicity (ADCC), present on 70 to 79% of CD70^-^ cells compared to 94 to 97% CD70^+^ cells) and CD158b^+^ (20 to 24% of CD70^-^ cells compared to 57 to 63% of CD70^+^ cells) was observed in CD70^+^ NK cells. Moreover, the gMFI of CD158b^+^ (KIR2DL2+) and NKp46^+^ cells were significantly higher in the CD8α^+^CD70^-^ subset compared to the CD8α^-^CD70^+^ subset. Finally, the gMFI of NKp30^+^ cells (a Natural Cytotoxicity activity receptor (NCR)) was significantly higher in the CD8α^+^CD70^+^ subset compared to the CD8α^-^CD70^-^ subset. Overall, at 16 days post-IL-2 expansion, differences in the expression of some activating and inhibitory receptors correlated to CD8α and CD70 expression were observed. However, there was no overarching phenotype related to the expression of CD8α or CD70, which would indicate a clear outcome for cytotoxicity.

### CD8α^+^CD70^-^ NK cell subsets are more cytotoxic against multiple target cells

**Figure 4:**
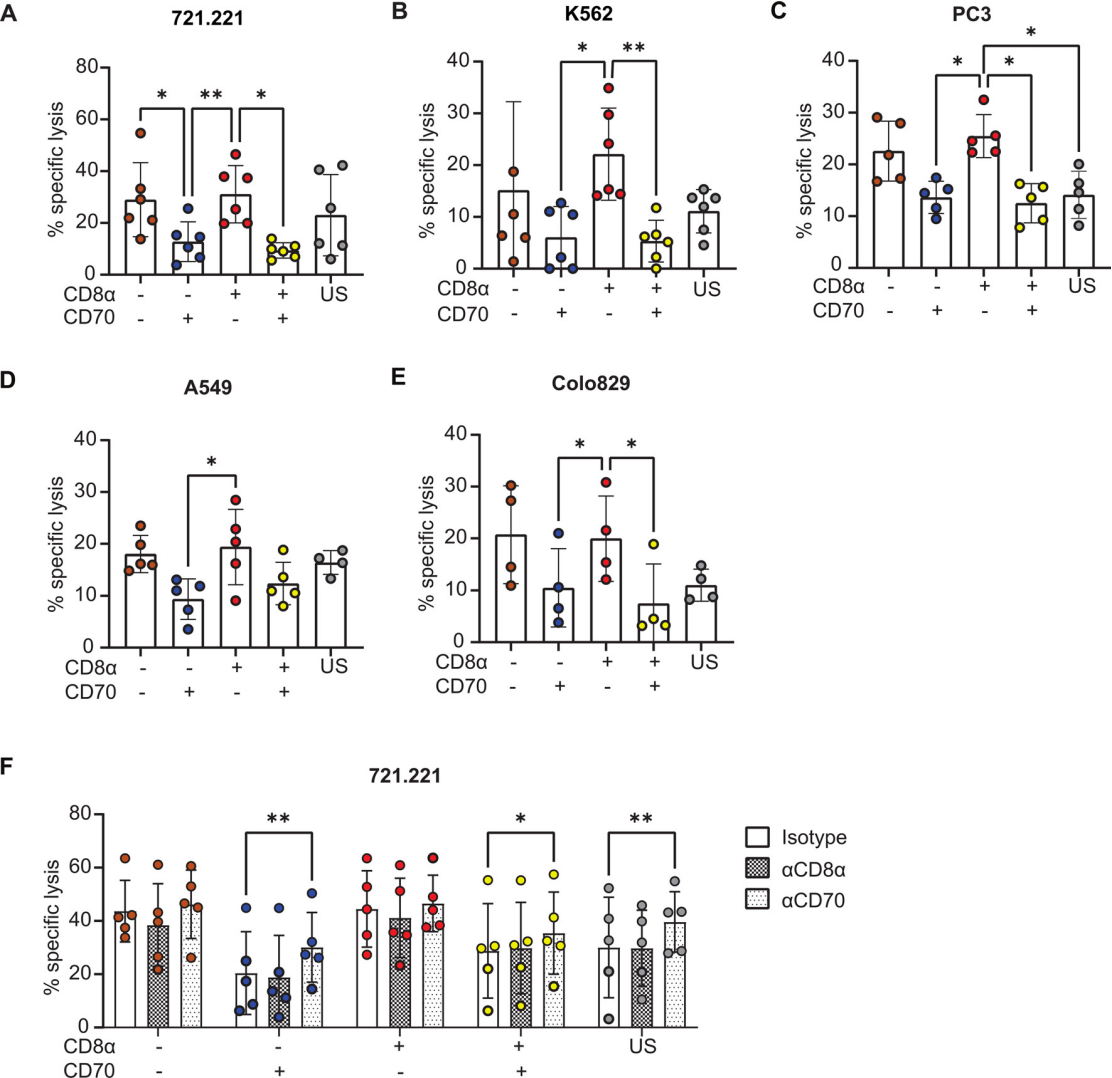
The CD8α^+^CD70^-^NK cell subset is particularly cytotoxic. **(A)** CD8α/CD70 subsets and unsorted (US) cytotoxicity profiles against 721.221 targets at 4h, at a 0.5:1 E:T ratio. Data from 6 individual donors. **(B–E)** CD8α/CD70 and US NK cell subset cytotoxicity profiles against K562, PC3, A549, Colo829 targets at 4h, at a 1:1 E:T ratio. Data from 6, 5, 5 and 4 individual donors, respectively. Mean and ± SD are indicated. **(A–E)** *P<0.05; **P<0.01, calculated by one-way ANOVA. **(F)** CD8α/CD70 and unsorted (US) NK cell subset cytotoxicity profiles against 721.221 targets, pre-incubated with isotype or blocking antibodies against CD8α and CD70. Data from 5 individual donors. Mean and ± SD are indicated. *P<0.05; **P<0.01, calculated by 2-way ANOVA.

Next, NK cells were sorted according to their expression of CD8α and CD70 and tested for their cytotoxicity against classical NK target cell lines 721.221 (B cell lymphoma) and K562 (myeloid leukaemia), as well as non-hematological tumor cell lines PC3 (prostate), A549 (lung) and Colo829 (melanoma). In all cases, CD8α^+^CD70^-^ population demonstrated strong killing; two to three-fold more cytotoxic than the CD8α^-^CD70^+^ population (**Figures 4A–E**). However, CD8α^+^CD70^-^ cells were not significantly more potent at killing than CD8α^-^CD70^-^ NK cells. Thus, most striking was that for all target cell lines, there was a significant and marked decrease in cytotoxicity for CD70^+^ NK cells (**Figures 4A–E**). The role of CD8α, however, was not evident in these bulk assays of cytotoxicity.

To test whether CD8α and CD70 proteins directly regulate cytotoxicity or simply mark subsets with differential cytotoxicity, we assessed whether blocking antibodies against these two proteins altered the killing of 721.221 target cells by NK cells. In line with previous work (38), blocking CD8α had no effect on killing (**Figure 4F**). In contrast, blocking CD70 partially restored killing in CD70^+^ subsets and increased the cytotoxicity of unsorted NK cell populations (**Figure 4F**). This suggests that CD70 can directly impact NK cell cytotoxicity.

### Time-lapse microscopy reveals that CD8α^+^CD70^-^ NK cells have a greater propensity to sequentially kill multiple target cells

Measurements of bulk levels of NK cell killing can lose nuances in the anti-tumor activity of NK cell subsets, not least because cells are brought into proximity, in tubes or wells, by gravity. In bulk assays, it is likely that single cell-cell interactions and one-on-one killing events are dominant. An alternative assessment of killing potential can be carried out using fluorescent time-lapse microscopy, which enables the visualization of NK cell killing dynamics and quantification of sequential interactions. Therefore, we next imaged the interactions of sorted populations of NK cells with 721.221 target cells.

**Figure 5:**
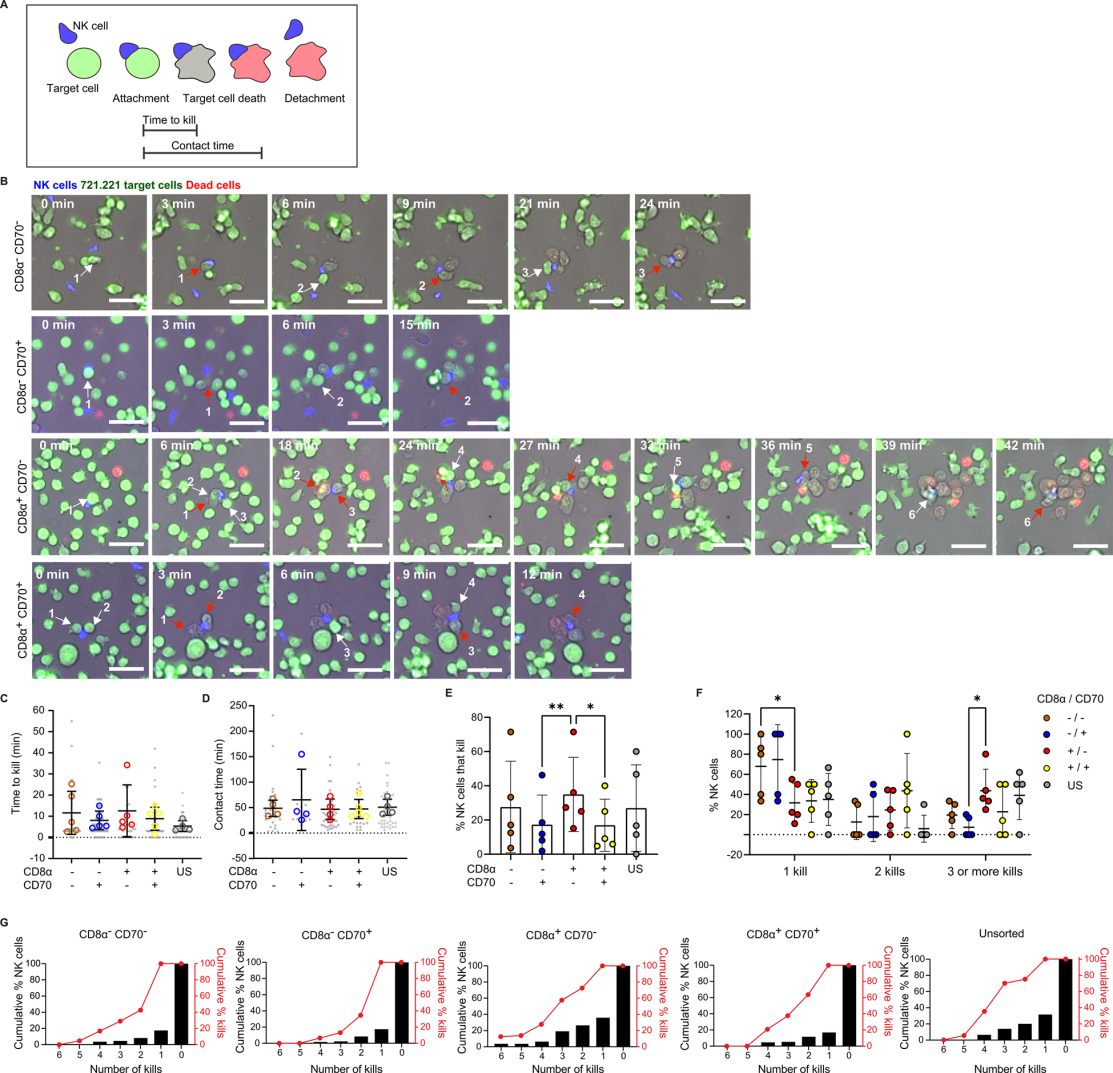
Time-lapse microscopy reveals CD8α^+^CD70^-^ NK cells to be especially good at sequentially killing multiple target cells. **(A)** Schematic of the interaction, colorimetric and morphological parameters that allow identification of target cell killing events by NK cells, as well as NK/target cell contact times and times to kill. **(B)** Representative time courses of serial killing events observed within each CD8α/CD70 NK cell subset. Sequentially killed 721.221 target cells are numbered incrementally and indicated by white arrows (frame prior to target cell death) and red arrows (frame when target cell death is detected). Scale bar 50 µm. **(C)** Time to kill target cells in the different CD8α/CD70 NK cell subsets and unsorted (US) NK cells. **(D)** Contact times between target cells and NK cells from the different CD8α/CD70 and US subsets. **(E)** Percentage of NK cells that kill. **(F)** Percentage of NK cells that kill one, two or three and more targets. **(G)** Cumulative percentage of NK cells that kill up to 6 target cells (black bars) and corresponding cumulative numbers of killing events (red dots and lines). **(C–G)** Data from cells derived from 5 individual healthy donors. The numbers of NK cells analyzed were 125, 134, 146, 159 and 122 for the populations of CD8α^+^CD70+, CD8α^+^CD70^-^ CD8α^-^CD70^+^ CD8α^-^CD70^-^ and unsorted cells, respectively **(C, D)** Each grey dot represents an individual cell, and each open circle represents the median of each individual donor. **(E, F)** Each open circle represents the proportion of cells that kill one, two or three or more target per donor. Mean and ± SD are indicated. *P<0.05; **P<0.01; **(C–E)** calculated by one-way ANOVA and **(F)** 2-way ANOVA.

To mimic the extracellular 3D matrix environment, Geltrex™ (a basement membrane matrix) was used to coat optically clear wells. To identify cell-cell interactions, NK cells and target cells were stained with calcein red-orange and calcein green, respectively. TOPRO-3™ was added to the supernatant to signal target cell death, as this dye fluoresces upon binding the DNA of dead cells. Images were acquired at 3-minute intervals for 5 hours to allow the identification of killing events, contact times and times to kill and determine the proportion of cells that are serial killers (**Figures 5A, B**; **Supplementary Videos 1**-**4**).

Subsets positive or negative for CD8α and CD70 displayed similar times to kill and a similar duration of contacts (**Figures 5C, D**). However, the proportion of CD8α^+^CD70^-^ NK cells that killed 721.221 target cells was significantly higher than in CD70^+^ subsets (**Figure 5E**), providing further evidence that CD70 impedes NK cell killing (**Figure 4**). Strikingly, CD8α^+^CD70^-^ NK cell subsets exhibited particularly potent serial killing. Significantly fewer CD8α^+^CD70^-^ NK cells killed only one target cell compared to the CD8α^-^CD70^-^ subset. Similarly, more CD8α^+^CD70^-^ NK cells killed three or more targets compared to the CD8α^-^CD70^+^ subset (**Figures 5F, G**). Cumulative analysis demonstrates that NK cells that killed at least 6 targets in CD8α^+^CD70^-^ subsets were responsible for 15% of the total kills. Together, these data provide evidence that CD8α expression marks an NK cell population with enhanced serial killing activity.

## Discussion

The ability of NK cells to identify and lyse cancer cells, as well as their potential for *ex vivo* expansion and adoptive transfer, makes them attractive candidates for immune therapies. In this work, we identified genes that are differentially regulated in NK cell clones according to their cytotoxic capacity. Upon culture with IL-2, cellular proliferation pathways were significantly associated with higher NK clone cytotoxicity at early times of culture (4 to 5 weeks). At later times of culture (2-5 months), transcriptional signatures of potent and weakly cytotoxic NK cell clones included high expression of *CD8Α* and *CD70*, respectively. Assessment of sorted NK cell populations revealed that expression of CD8α is associated with high levels of serial killing, while CD70 plays a role in the inhibition of NK cell cytotoxicity.

Our transcriptomic data uncovered an upregulation of cellular proliferation genes in the most cytotoxic clones after 4 to 5 weeks of culture. As IL-2 is well-established to stimulate proliferation and cytotoxicity of NK cells (39), it is possible that this finding relates to some NK cells responding particularly well to IL-2, although IL-2 receptor gene expression did not vary with cytotoxicity in our experiments. Surprisingly, an increase in *IL15* expression was detected in the least cytolytic NK clones. IL-15 is a cytokine that signals through components of the IL-2 receptor and can stimulate NK cells in a similar manner to IL-2 (40). Existing publications have demonstrated that IL-15 can induce NK cells into a hypo-responsive state or exhaustion upon prolonged exposure (41, 42). Thus, poorly cytotoxic NK clones may relate to this phenotype. Many other genes (such as *H2AZ1* and *COTL1*) were upregulated in ‘best’ versus ‘worst’ killer NK clones after short-term culture and warrant further investigation.

At later times of culture, a distinct transcriptional signature was associated with NK cell cytotoxicity. Although cytotoxicity levels were diminished at this time of culture, lysis of target cells remained at least 7-fold higher in the best killers, compared to the worst killers. In potent NK cell clones, *CD8A* was the most significantly enriched mRNA. This gene encodes CD8α, a component of the CD8 receptor typically expressed at the surface of cytotoxic T cells, which acts as a co-receptor with the T-cell receptor and directly binds MHC-class I proteins (43–45). Recent publications have implicated a role for CD8 in modulating NK cell activation and have identified CD8 as a marker of NK cells with enhanced proliferative fitness (46). Moreover, a previous study described a role for CD8α in enhancing NK cell cytotoxicity by preventing activation-induced apoptosis (38). Here, CD8α blockade did not affect cytotoxicity directly; however, anti-CD8 antibodies vary in the epitopes that they target, and it is unclear which CD8α interactions are at play in NK cells (47, 48). Using time-lapse microscopy, we observed that CD8α^+^ NK cells were better than CD8α^-^ NK cells at serial killing of 721.221 target cells (which are transformed cells selected to lack expression of class I MHC protein). It has been suggested that fraternal NK cell-NK cell interactions via CD8α on one NK cell and class I MHC protein on another NK cell can protect NK cells from activation-induced cell death, allowing them to kill more targets (38). However, we did not detect fraternal NK-NK interactions in our time-lapse microscopy experiments. It is therefore likely that CD8α expression on NK cells can also impact serial killing via an alternative mechanism, either directly or as a marker for a subset of cells adept at serial killing.

CD70 belongs to the TNF family and is the selective ligand for CD27 (49). CD70 is transiently expressed on lymphocytes following activation and can mediate ‘reverse-signaling’ (50). It has been implicated in better responsiveness of NK cells to CD27 expressing malignancies (51). Here, we found that expression of CD70 on NK cells tended to increase over time in the presence of IL-2. Cytotoxicity assays revealed that expression of CD70 on NK cells was associated with impaired killing. Treatment of CD70^+^ NK cells with an anti-CD70 blocking mAb was found to enhance killing. Thus, CD70 may act as an immune checkpoint molecule in NK cells. The role of CD70 is also likely to be important in T cells, as knocking out CD70 has been shown to improve CAR T cell function (52).

Overall, our approach to analyzing genes expressed in NK clones with differential cytotoxicity has identified several novel determinants of NK cell killing. As well as improving our fundamental understanding of NK cell cytotoxicity, these data could be used to define optimal subsets of NK cells for immune therapies, identify proteins which could be induced *ex vivo* to improve function or identify new molecular targets for genetic knockout or antibody blockade. For example, the development of single guide RNAs to knockout CD70 may be worth exploring to circumvent NK cell inhibition and dysregulation. More broadly, our strategy of analyzing clones according to specific functions could be utilized to identify avenues to enhance immune cell responses in next-generation immune therapies.

## Materials and methods

### Cell culture

#### Cell lines

All cell lines were of human origin and maintained at 37°C and 5% CO_2_. Target cell lines 721.221 (transformed B cell), K562 (myelogenous leukemia), Colo829 (melanoma) and feeder cells RPMI 8866 (myelogenous leukemia) were grown in Roswell Park Memorial Institute (RPMI)-1640 (Sigma) containing 10% heat-inactivated FBS (Gibco), 2 mM L-Glutamine (Sigma) and 50 U/ml Penicillin/Streptomycin (Sigma). Target cell lines PC3 (prostate cancer) and A549 (alveolar basal adenocarcinoma) were grown in Ham’s F-12 media (Sigma) containing 10% heat-inactivated FBS (Gibco) and 50 U/ml Penicillin/Streptomycin (Sigma). Cell lines were maintained through passage every 3-4 days. All cell lines were routinely tested for mycoplasma infection using a PCR-based kit (PromoCell).

#### Isolation of primary human NK cells

Whole blood of healthy donors was obtained from the National Blood Transfusion Service (Manchester, UK), following the appropriate ethics guidelines (License: 05/Q0401/108). Alternatively, blood samples from Long-Term Cancer Survivors were used (Continuum Life Sciences) via the Swansea University Continuum Biobank, as approved by the SU CTB Biobank Scientific and Ethical Review Committee (Ref: SU_CTBB003). Blood samples from three Long-Term Cancer Survivors (Continuum Life Sciences) were used in this study. Donors 1 and 2 were treatment free at the time of the study. Donor 1 had been in remission for 9 years from breast and colorectal cancer, while donor 2 had been in remission from colorectal cancer for 15 years. Donor 3 was undergoing treatments that haven’t been directly linked to the immune system (antibiotics, pancreatin, codeine, probiotics, anti-nausea medication) and had been in remission for 5 years from colorectal, pancreatic, liver and basal cell carcinoma cancers.

Whole blood was diluted in Phenol-red free RMPI and layered upon Ficoll-Paque™ (GE Healthcare) in a 50ml Falcon tube. Peripheral blood mononuclear cells (PBMC’s) were isolated using density gradient centrifugation at 1600 RPM for 40 minutes with no break upon deceleration. The buffy coat was then collected and the PBMC’s washed twice in Phenol-red free RMPI. PBMC’s derived from cones were resuspended in Red Blood Cell Lysing buffer (Sigma) and incubated for 5 minutes at room temperature and washed as before with Phenol-red free RMPI to stop the reaction. NK cells were negatively selected from PBMC’s using an NK cell isolation kit (MiltenyiBiotec). Once isolated, NK cells were cultured in clone medium: DMEM (Gibco) supplemented with 30% F-12 Ham (Gibco), 10% Human Serum (Sigma), 1% Non-essential amino acids (Sigma), 1mM Sodium pyruvate (Sigma), 2 mM L-Glutamine (Sigma), 50 U/ml Penicillin/Streptomycin and 50 µM β-Mercaptoethanol (Gibco) containing 200U/ml IL-2 (Roche). NK cells were used in experiments 6 days post-isolation, unless otherwise stated.

#### Generation of NK cell clones

NK cell clones were isolated from polyclonal populations of primary NK cells using serial dilution or cell sorting. Prior to clone generation, feeder cells (2.5x10^6^ RMPI 8866 (Sigma) and 100×10^6^ allogeneic PBMCs obtained from two different donors) were irradiated with 2x20 gray (Gy), to supplement 50ml of clone media. Clones were derived by serially diluting NK cells from 10^3^ cells/ml to 3 cells/ml in clone media supplemented with irradiated feeder cells, PHA (5μg/ml; Thermo Scientific) and 400U/ml IL-2. To each well of a 96 well U-bottom plate, 100 µl of the cells in complete medium were added to plate the clones at a concentration of 0.3 cells/well. Alternatively, NK cells were stored overnight at 4°C in MACs buffer (PBS containing 0.5% BSA and 2mM EDTA), for sorting the following day. NK cells were washed in FACS buffer (PBS containing 1% FBS) and stained using anti-CD3 PE (Biolegend) and anti-CD56 FITC (Biolegend) for 30 minutes at 4°C. Cells were then washed with FACS buffer before cell sorting (BD Influx). CD3-negative and CD56-positive cells were gated and single-cell sorted into a 96-well U-bottom plate containing clone media supplemented with irradiated feeder cells, 5ug/ml PHA and 400U/ml IL-2. Once plated, NK cell clones were incubated at 37°C and 5% CO_2_ and re-stimulated 7 days later with clone medium containing irradiated feeder cells, 5μg/ml PHA and 400U/ml IL-2. Expanded clones were transferred to 48-well plates then later expanded into 24-well plates and cultured in fresh clone medium containing 200U/ml every 5-6 days.

### Flow cytometry

To analyze the expression of extracellular markers, 5x10^4^-1x10^5^ cells were washed in FACS tubes by adding PBS and centrifuging at 1300rpm at 4°C for 5 minutes and stained with LIVE/DEAD™ Fixable blue viability dye (Invitrogen). Cells were then washed with FACS buffer (PBS with 2% BSA) and blocked with 2% human serum (Sigma) at 4°C for 15 minutes (to prevent non-specific binding through Fcγ receptors). Following this, primary antibodies (full details in **Supplementary Table 1**) or isotype-matched controls were then added directly to the tubes and incubated for a further 30 minutes at 4°C and then washed with FACS buffer. Alternatively, for clone screening, 50,000-100,000 cells were pelleted in a 96 well V-bottom plate and washed in 150 µl PBS. Pellets were resuspended in 50ul of PBS containing LIVE/DEAD™ Fixable Blue dead cell membrane dye and incubated at 4°C for 10 minutes. The cells were then washed with 100 µl of FACS buffer containing 2% human serum at 4°C for 15 minutes. The primary antibodies or isotype-matched controls were then added directly to the wells and incubated for a further 30 minutes at 4°C. Cells were then fixed in 2% PFA, stored at 4°C and analyzed by flow cytometry (FACS Symphony or LSR Fortessa SORP, BD). All flow cytometry analysis was carried out using FlowJo V10.

### Functional assays

#### Annexin-V/Propridium Iodide cytotoxicity assay

NK cell cytotoxicity was analyzed by staining for phosphatidylserine exposure using Annexin-V APC (BioLegend) and membrane permeability using PI (Thermofisher). One day prior to the experiment, target cells were seeded in fresh complete RMPI media. The next day, to make target cells distinguishable from NK cells, NK cells were washed and resuspended at 1x10^6^ cells/ml in serum-free media and labelled using CellTrace™ Violet (Invitrogen) as per the manufacturer’s instructions. Labelled NK cells were then washed and combined with target cells in complete media in a V-bottom 96-well plate, at the appropriate effector to target ratio or 0:1 (to control for spontaneous lysis) in a total of 100 µl of media and incubated at 37°C and 5% CO₂ for 4 hours. Cells were then washed with Annexin-V binding buffer (25mM CaCl2 + 1.4M NaCl, +0.1M HEPES) and incubated with 2μl of Annexin-V at room temperature for 10 minutes. The cells were then washed with Annexin-V binding buffer and stained with 1μg/ml of PI and kept on ice until analysis on the BD LSR Fortessa X20. All samples were analyzed within an hour of assay completion. To calculate the percentage of specific lysis for each sample, the following calculation was used:


$$\begin{array}{c} \text{\% }\;Specific\;target\;lysis=\\ \text{\% }target\;lysis-\text{\% }spontaneous\;lysis \end{array}$$

When performing blocking assays, NK cells were labelled as described before using CellTrace™ Violet. They were then incubated with isotype IgG1k, blocking anti-CD8Α antibody (SK1 clone, Biolegend) or anti-CD70 antibody (clone BU69, Abcam) at 10 µg/mL for 1 hour at 37°C. The NK cells were then washed and combined to 721.221 target cells, at a 1:1 E:T ratio. The following procedure was performed as described for standard cytotoxicity assays.

#### Radioactive release cytotoxicity assay

NK cell cytotoxicity was determined by measuring the release of S-35 upon lysis of radio-labeled target cells. One day prior to the experiment, target cells were irradiated with 100µCi S-35. The next day, irradiated target cells were washed three times in serum-free media and resuspended at 1x10^5^ cells/ml. The target cells were then combined with NK cells in a V-bottom 96-well plate, at an effector to target ratio of 5:1 or 0:1 (to control for spontaneous lysis) or 0:1 in the presence of 0.05% Triton X-100 (to measure maximum lysis), in a total of 100 µl media and incubated at 37°C and 5% CO_2_ for 4 hours. The killing assay was then stopped by pelleting the cells at 4°C and supernatants were collected and combined with scintillation liquid (Microscint 40, Perkin Elmer) in 96 well optiplates overnight on a shaker. Radioactive release was read the following day using a radiometric plate reader (Microbeta2, Perkin Elmer). To calculate the percentage of specific lysis for each sample, the following calculation was used:


$$\begin{array}{c} \text{\% }\;specific\;target\;lysis=\\ \frac{(\text{\% sample}\;\text{lysis}-\text{\% spontaneous}\;\text{lysis})}{(\text{\% maximum}\;\text{lysis}-\text{\% spontaneous}\;\text{lysis})}\times 100 \end{array}$$

#### IFN-γ ELISA

Wells of a Nunc-Immuno™ flat-bottom 96-well plate (Thermo Scientific) were coated with 50µl of 0.01% Poly-L-Lysine (Sigma) and incubated at 37°C for 10 minutes. Following the removal of the solution, wells were washed three times using sterile water, before being dried at 60°C for 1 hour. Individual wells were coated with ICAM alone or in combination with Fc-MICA (R&D Systems and Manchester Institute of Biotechnology, respectively) diluted in 50µl PBS, and incubated at 4°C overnight. Alternatively, wells prepared for unstimulated controls were coated in PBS only. The next day, the solution was removed, and the wells were washed three times with PBS in prior to the addition of NK cells. For all stimulatory conditions, 10^5^ NK cells were resuspended in 100µl of fresh clone medium added to each well and incubated overnight at 37°C and 5% CO₂. Supernatants were then centrifuged at 1300rpm at 4°C for 10 minutes in a V-bottom 96 well plate and transferred to a new flat-bottom 96 well plate for storage at -20°C. Secretion of IFN-γ was then quantified using the DuoSet ELISA kit (R&D Systems), as per the manufacturer’s instructions.

#### Time-lapse microscopy assay

NK cells and target cells were washed and stained with 0.64 µM calcein red-orange and 0.3 µM calcein green, respectively, in serum-free media for 20 minutes, at 37°C in 5% CO₂. In parallel, the wells of optically clear 96 well plates (Greiner) were coated with a thin layer of Geltrex™ (Thermofisher) 30 minutes at 37°C. Fluorescently labelled NK cells and target cells were washed and brought to 200,000 and 800,000 cells/mL, respectively, in complete media. 50 µl of both cellular suspensions were then added to the wells, in the presence of 2% Geltrex™ and 1µM To-pro-3 (Thermofisher). Images were acquired immediately by widefield microscopy (Nikon Eclipse Ti) at 3-minute intervals for 5 hours at 37°C, 5% CO₂. A 10x/0.45 N.A. or 0.3 N.A. air objective was used with the following excitation/emission filter sets: 395/460nm, 470/525nm and 570/645nm and pE-300 LED (CoolLED) fluorescent light sources. Images were analyzed using Image-J.

### RNA isolation and analysis

#### RNA extraction

To obtain sufficient RNA for sequencing, 0.65-1x10^6^ cells were resuspended in 350 µl RTL buffer containing 50 µM β-mercaptoethanol, vortexed thoroughly and stored at -80°C prior to extraction. RNA was extracted from thawed using the RNeasy^®^ Mini Kit (Qiagen) as per the manufacturer’s instructions. To prevent saturation, a maximum of 0.5x10^6^ cells were loaded into each extraction column. Following extraction, RNA concentrations were measured using the Nanodrop™ spectrophotometer.

#### RNA sequencing

For each clone, 1μg RNA was submitted for next-generation sequencing at the University of Manchester Genomic Technologies Core Facility. Libraries were prepared using the Illumina stranded mRNA prep kit, labelled using dual unique barcodes and pooled prior to loading onto the Illumina HiSeq 4000 system.

### Statistical analysis

GraphPad Prism 9 was used to plot and statistically analyze all functional datasets. Prior to analysis, data sets were tested for normality using a Shapiro-Wilk normality test. Following this a parametric or non-parametric test was performed for normally distributed and non-normally distributed data, respectively. Results are expressed as the mean ± standard deviation (SD). Statistical significance was determined when P ≤ 0.05 (*) P ≤ 0.01 (**), P ≤ 0.001 (***) and P ≤ 0.0001 (****). When P>0.05, no significant differences (ns) were determined.

FlowJo v10 was used to perform a tSNE dimensional reduction on a concatenated population of equal numbers of CD70^+^ CD8α^-^, CD70^+^ CD8α^+^, CD70^-^ CD8α^+^, CD70^-^CD8α^-^ sorted NK cells, based on their expression of NKp30, CD16, CD56, CD158b, CD27, TIGIT, NKG2D, CD3 and NKp46. The four populations defined by their expression of CD70 and CD8 were then plotted separately in the previously determined tSNE space and colored for expression of the analyzed markers (High expression red to low expression blue). X-shift unsupervised clustering was used as an unbiased method to identify populations (53).

The RNA sequencing data analysis was performed in collaboration with the Genomics Technology Core Facility, using the DESeq2 package (54). The resulting datasets were then analyzed using R to produce Volcano plots, gProfiler for gene ontology analysis on ranked DE genes and GenePattern for unranked GSEA analysis of DE genes (29).

## Data Availability

The data presented in this publication have been deposited in NCBI's Gene Expression Omnibus (55) and are accessible through GEO Series accession number GSE289212 (https://www.ncbi.nlm.nih.gov/geo/query/acc.cgi?acc=GSE289212).

## References

[1] Davis DM Chiu I Fassett M Cohen GB Mandelboim O Strominger JL. The human natural killer cell immune synapse. Proc Natl Acad Sci U.S.A. (1999) 96:15062–7. doi: 10.1073/pnas.96.26.15062 PMC24773 10611338

[2] Moretta L Bottino C Pende D Castriconi R Mingari MC Moretta A. Surface NK receptors and their ligands on tumor cells. Semin Immunol. (2006) 18:151–8. doi: 10.1016/j.smim.2006.03.002 16730454

[3] Vivier E Raulet DH Moretta A Caligiuri MA Zitvogel L Lanier LL. Innate or adaptive immunity? The example of natural killer cells. Science. (2011) 331:44–9. doi: 10.1126/science.1198687 PMC3089969 21212348

[4] Freud AG Mundy-Bosse BL Yu JH Caligiuri MA. The broad spectrum of human natural killer cell diversity. Immunity. (2017) 47:820–33. doi: 10.1016/j.immuni.2017.10.008 PMC5728700 29166586

[5] Smith SL Kennedy PR Stacey KB Worboys JD Yarwood A Seo S. Diversity of peripheral blood human NK cells identified by single-cell RNA sequencing. Blood Adv. (2020) 4:1388–406. doi: 10.1182/bloodadvances.2019000699 PMC7160259 32271902

[6] Crinier A Milpied P Escalière B Piperoglou C Galluso J Balsamo A. High-dimensional single-cell analysis identifies organ-specific signatures and conserved NK cell subsets in humans and mice. Immunity. (2018) 49:971–86. doi: 10.1016/j.immuni.2018.09.009 PMC6269138 30413361

[7] Horowitz A Strauss-Albee DM Leipold M Kubo J Nemat-Gorgani N Dogan OC. Genetic and environmental determinants of human NK cell diversity revealed by mass cytometry. Sci Transl Med. (2013) 5:208ra145. doi: 10.1126/scitranslmed.3006702 PMC3918221 24154599

[8] Rebuffet L Melsen JE Escalière B Basurto-Lozada D Bhandoola A Björkström NK. High-dimensional single-cell analysis of human natural killer cell heterogeneity. Nat Immunol. (2024) 25:1474–88. doi: 10.1038/s41590-024-01883-0 PMC11291291 38956378

[9] Vanherberghen B Olofsson PE Forslund E Sternberg-Simon M Khorshidi MA Pacouret S. Classification of human natural killer cells based on migration behavior and cytotoxic response. Blood. (2013) 121:1326–34. doi: 10.1182/blood-2012-06-439851 23287857

[10] Prager I Liesche C van Ooijen H Urlaub D Verron Q Sandström N. NK cells switch from granzyme B to death receptor–mediated cytotoxicity during serial killing. J Exp Med. (2019) 216:2113–27. doi: 10.1084/jem.20181454 PMC6719417 31270246

[11] Barrow AD Martin CJ Colonna M. The natural cytotoxicity receptors in health and disease. Front Immunol. (2019) 10:909. doi: 10.3389/fimmu.2019.00909 31134055 PMC6514059

[12] Schmied L Luu TT Søndergaard JN Hald SH Meinke S Mohammad DK. SHP-1 localization to the activating immune synapse promotes NK cell tolerance in MHC class I deficiency. Sci Signaling. (2023) 16:eabq0752. doi: 10.1126/scisignal.abq0752 37040441

[13] Simonetta F Alvarez M Negrin RS. Natural killer cells in graft-versus-host-disease after allogeneic hematopoietic cell transplantation. Front Immunol. (2017) 8:465. doi: 10.3389/fimmu.2017.00465 28487696 PMC5403889

[14] Tavaf MJ Verkiani ME Hanzaii FP Zomorrod MS. Effects of immune system cells in GvHD and corresponding therapeutic strategies. Blood Res. (2023) 58:2–12. doi: 10.5045/br.2023.2022192 36774947 PMC10063589

[15] Laskowski TJ Biederstädt A Rezvani K. Natural killer cells in antitumour adoptive cell immunotherapy. Nat Rev Cancer. (2022) 22:557–75. doi: 10.1038/s41568-022-00491-0 PMC9309992 35879429

[16] Berrien-Elliott MM Jacobs MT Fehniger TA. Allogeneic natural killer cell therapy. Blood. (2023) 141:856–68. doi: 10.1182/blood.2022016200 PMC10023727 36416736

[17] Robertson MJ Manley TJ Donahue C Levine H Ritz J. Costimulatory signals are required for optimal proliferation of human natural killer cells. J Immunol. (1993) 150:1705–14. doi: 10.4049/jimmunol.150.5.1705 7679691

[18] Valiante NM Uhrberg M Shilling HG Lienert-Weidenbach K Arnett KL D'Andrea A. Functionally and structurally distinct NK cell receptor repertoires in the peripheral blood of two human donors. Immunity. (1997) 7:739–51. doi: 10.1016/s1074-7613(00)80393-3 9430220

[19] Yang C Siebert JR Burns R Gerbec ZJ Bonacci B Rymaszewski A. Heterogeneity of human bone marrow and blood natural killer cells defined by single-cell transcriptome. Nat Commun. (2019) 10:3931. doi: 10.1038/s41467-019-11947-7 31477722 PMC6718415

[20] Berg M Lundqvist A McCoy P Jr. Samsel L Fan Y Tawab A. Clinical-grade ex vivo-expanded human natural killer cells up-regulate activating receptors and death receptor ligands and have enhanced cytolytic activity against tumor cells. Cytotherapy. (2009) 11:341–55. doi: 10.1080/14653240902807034 PMC2736058 19308771

[21] Wolf NK Kissiov DU Raulet DH. Roles of natural killer cells in immunity to cancer, and applications to immunotherapy. Nat Rev Immunol. (2023) 23:90–105. doi: 10.1038/s41577-022-00732-1 35637393

[22] Streltsova MA Erokhina SA Kanevskiy LM Lee DA Telford WG Sapozhnikov AM. Analysis of NK cell clones obtained using interleukin-2 and gene-modified K562 cells revealed the ability of “senescent” NK cells to lose CD57 expression and start expressing NKG2A. PLoS One. (2018) 13:e0208469. doi: 10.1371/journal.pone.0208469 30517188 PMC6281266

[23] Sconocchia G Titus JA Mazzoni A Visintin A Pericle F Hicks SW. CD38 triggers cytotoxic responses in activated human natural killer cells. Blood. (1999) 94:3864–71. doi: 10.1182/blood.V94.11.3864 10572102

[24] Lee J Robinson ME Ma N Artadji D Ahmed MA Xiao G. IFITM3 functions as a PIP3 scaffold to amplify PI3K signaling in B cells. Nature. (2020) 588:491–7. doi: 10.1038/s41586-020-2884-6 PMC8087162 33149299

[25] Oyer HM Sanders CM Kim FJ. Small-molecule modulators of sigma1 and sigma2/TMEM97 in the context of cancer: foundational concepts and emerging themes. Front Pharmacol. (2019) 10:1141. doi: 10.3389/fphar.2019.01141 31695608 PMC6816035

[26] Kim J Shapiro MJ Bamidele AO Gurel P Thapa P Higgs HN. Coactosin-like 1 antagonizes cofilin to promote lamellipodial protrusion at the immune synapse. PLoS One. (2014) 9:e85090. doi: 10.1371/journal.pone.0085090 24454796 PMC3890291

[27] Martín-Cófreces NB Sánchez-Madrid F. Sailing to and docking at the immune synapse: role of tubulin dynamics and molecular motors. Front Immunol. (2018) 9:1174. doi: 10.3389/fimmu.2018.01174 29910809 PMC5992405

[28] Kim W Fan Y-Y Barhoumi R Smith R McMurray DN Chapkin RS. n-3 polyunsaturated fatty acids suppress the localization and activation of signaling proteins at the immunological synapse in murine CD4+ T cells by affecting lipid raft formation1. J Immunol. (2008) 181:6236–43. doi: 10.4049/jimmunol.181.9.6236 PMC2597670 18941214

[29] Reimand J Isserlin R Voisin V Kucera M Tannus-Lopes C Rostamianfar A. Pathway enrichment analysis and visualization of omics data using g:Profiler, GSEA, Cytoscape and EnrichmentMap. Nat Protoc. (2019) 14:482–517. doi: 10.1038/s41596-018-0103-9 30664679 PMC6607905

[30] Granzin M Wagner J Köhl U Cerwenka A Huppert V Ullrich E. Shaping of natural killer cell antitumor activity by ex vivo cultivation. Front Immunol. (2017) 8:458. doi: 10.3389/fimmu.2017.00458 28491060 PMC5405078

[31] Shao JY Yin WW Zhang QF Liu Q Peng ML Hu HD. Siglec-7 defines a highly functional natural killer cell subset and inhibits cell-mediated activities. Scand J Immunol. (2016) 84:182–90. doi: 10.1111/sji.12455 27312286

[32] Wilson JL Charo J Martín-Fontecha A Dellabona P Casorati G Chambers BJ. NK cell triggering by the human costimulatory molecules CD80 and CD86. J Immunol. (1999) 163:4207–12. doi: 10.4049/jimmunol.163.8.4207 10510357

[33] Tata A Dodard G Fugère C Leget C Ors M Rossi B. Combination blockade of KLRG1 and PD-1 promotes immune control of local and disseminated cancers. Oncoimmunology. (2021) 10:1933808. doi: 10.1080/2162402x.2021.1933808 34188973 PMC8208121

[34] Müller-Durovic B Lanna A Covre LP Mills RS Henson SM Akbar AN. Killer cell lectin-like receptor G1 inhibits NK cell function through activation of adenosine 5'-monophosphate-activated protein kinase. J Immunol. (2016) 197:2891–9. doi: 10.4049/jimmunol.1600590 PMC5027915 27566818

[35] Torres A Tang A Vyas R Su C Johnson M Wells A. PAK6- A novel kinase in the maintenance of T cell anergy. J Immunol. (2019) 202:184.8–.8. doi: 10.4049/jimmunol.202.Supp.184.8

[36] Bradley T Peppa D Pedroza-Pacheco I Li D Cain DW Henao R. RAB11FIP5 expression and altered natural killer cell function are associated with induction of HIV broadly neutralizing antibody responses. Cell. (2018) 175:387–99.e17. doi: 10.1016/j.cell.2018.08.064 30270043 PMC6176872

[37] Chambers BJ Salcedo M Ljunggren HG. Triggering of natural killer cells by the costimulatory molecule CD80 (B7-1). Immunity. (1996) 5:311–7. doi: 10.1016/s1074-7613(00)80257-5 8885864

[38] Addison EG North J Bakhsh I Marden C Haq S Al-Sarraj S. Ligation of CD8alpha on human natural killer cells prevents activation-induced apoptosis and enhances cytolytic activity. Immunology. (2005) 116:354–61. doi: 10.1111/j.1365-2567.2005.02235.x PMC1802415 16236125

[39] Uharek L Zeis M Glass B Steinmann J Dreger P Gassmann W. High lytic activity against human leukemia cells after activation of allogeneic NK cells by IL-12 and IL-2. Leukemia. (1996) 10:1758–64. 8892679

[40] Carson WE Giri JG Lindemann MJ Linett ML Ahdieh M Paxton R. Interleukin (IL) 15 is a novel cytokine that activates human natural killer cells via components of the IL-2 receptor. J Exp Med. (1994) 180:1395–403. doi: 10.1084/jem.180.4.1395 PMC2191697 7523571

[41] Elpek KG Rubinstein MP Bellemare-Pelletier A Goldrath AW Turley SJ. Mature natural killer cells with phenotypic and functional alterations accumulate upon sustained stimulation with IL-15/IL-15Ralpha complexes. Proc Natl Acad Sci U.S.A. (2010) 107:21647–52. doi: 10.1073/pnas.1012128107 PMC3003106 21098276

[42] Felices M Lenvik AJ McElmurry R Chu S Hinderlie P Bendzick L. Continuous treatment with IL-15 exhausts human NK cells via a metabolic defect. JCI Insight. (2018) 3. doi: 10.1172/jci.insight.96219 PMC5821201 29415897

[43] Blue ML Craig KA Anderson P Branton KR Jr. Schlossman SF. Evidence for specific association between class I major histocompatibility antigens and the CD8 molecules of human suppressor/cytotoxic cells. Cell. (1988) 54:413–21. doi: 10.1016/0092-8674(88)90204-8 2969292

[44] Norment AM Salter RD Parham P Engelhard VH Littman DR. Cell-cell adhesion mediated by CD8 and MHC class I molecules. Nature. (1988) 336:79–81. doi: 10.1038/336079a0 3263576

[45] Rosenstein Y Ratnofsky S Burakoff SJ Herrmann SH. Direct evidence for binding of CD8 to HLA class I antigens. J Exp Med. (1989) 169:149–60. doi: 10.1084/jem.169.1.149 PMC2189183 2462606

[46] Cubitt CC Wong P Dorando HK Foltz JA Tran J Marsala L. Induced CD8α identifies human NK cells with enhanced proliferative fitness and modulates NK cell activation. J Clin Invest. (2024) 134. doi: 10.1172/jci173602 PMC11291271 38805302

[47] Clement M Pearson JA Gras S van den Berg HA Lissina A Llewellyn-Lacey S. Targeted suppression of autoreactive CD8+ T-cell activation using blocking anti-CD8 antibodies. Sci Rep. (2016) 6:35332. doi: 10.1038/srep35332 27748447 PMC5066216

[48] Wooldridge L Hutchinson SL Choi EM Lissina A Jones E Mirza F. Anti-CD8 antibodies can inhibit or enhance peptide-MHC class I (pMHCI) multimer binding: this is paralleled by their effects on CTL activation and occurs in the absence of an interaction between pMHCI and CD8 on the cell surface. J Immunol. (2003) 171:6650–60. doi: 10.4049/jimmunol.171.12.6650 14662868

[49] Hintzen RQ Lens SM Beckmann MP Goodwin RG Lynch D van Lier RA. Characterization of the human CD27 ligand, a novel member of the TNF gene family. J Immunol. (1994) 152:1762–73. doi: 10.4049/jimmunol.152.4.1762 8120385

[50] García P De Heredia AB Bellón T Carpio E Llano M Caparrós E. Signalling via CD70, a member of the TNF family, regulates T cell functions. J Leukoc Biol. (2004) 76:263–70. doi: 10.1189/jlb.1003508 15226368

[51] Al Sayed MF Ruckstuhl CA Hilmenyuk T Claus C Bourquin JP Bornhauser BC. CD70 reverse signaling enhances NK cell function and immunosurveillance in CD27-expressing B-cell Malignancies. Blood. (2017) 130:297–309. doi: 10.1182/blood-2016-12-756585 28495792

[52] Dequeant M-L Sagert J Kalaitzidis D Yu H Porras A McEwan B. CD70 Knockout: A novel approach to augment CAR-T cell function. Cancer Res. (2021) 81(13_Supplement):1537. doi: 10.1158/1538-7445.AM2021-1537

[53] Samusik N Good Z Spitzer MH Davis KL Nolan GP. Automated mapping of phenotype space with single-cell data. Nat Methods. (2016) 13:493–6. doi: 10.1038/nmeth.3863 PMC4896314 27183440

[54] Love MI Huber W Anders S. Moderated estimation of fold change and dispersion for RNA-seq data with DESeq2. Genome Biol. (2014) 15:550. doi: 10.1186/s13059-014-0550-8 25516281 PMC4302049

[55] Edgar R Domrachev M Lash AE. Gene Expression Omnibus: NCBI gene expression and hybridization array data repository. Nucleic Acids Res. (2002) 30:207–10. doi: 10.1093/nar/30.1.207 PMC99122 11752295

