# Supplementary material for: CD8α and CD70 mark human natural killer cell populations which differ in cytotoxicity
Source: Front Immunol. 2025 Feb 19;16:1526379. doi: 10.3389/fimmu.2025.1526379 (PMC11880019; doi:10.3389/fimmu.2025.1526379)
Supplement: Supplementary file 1 [file Image1.pdf]

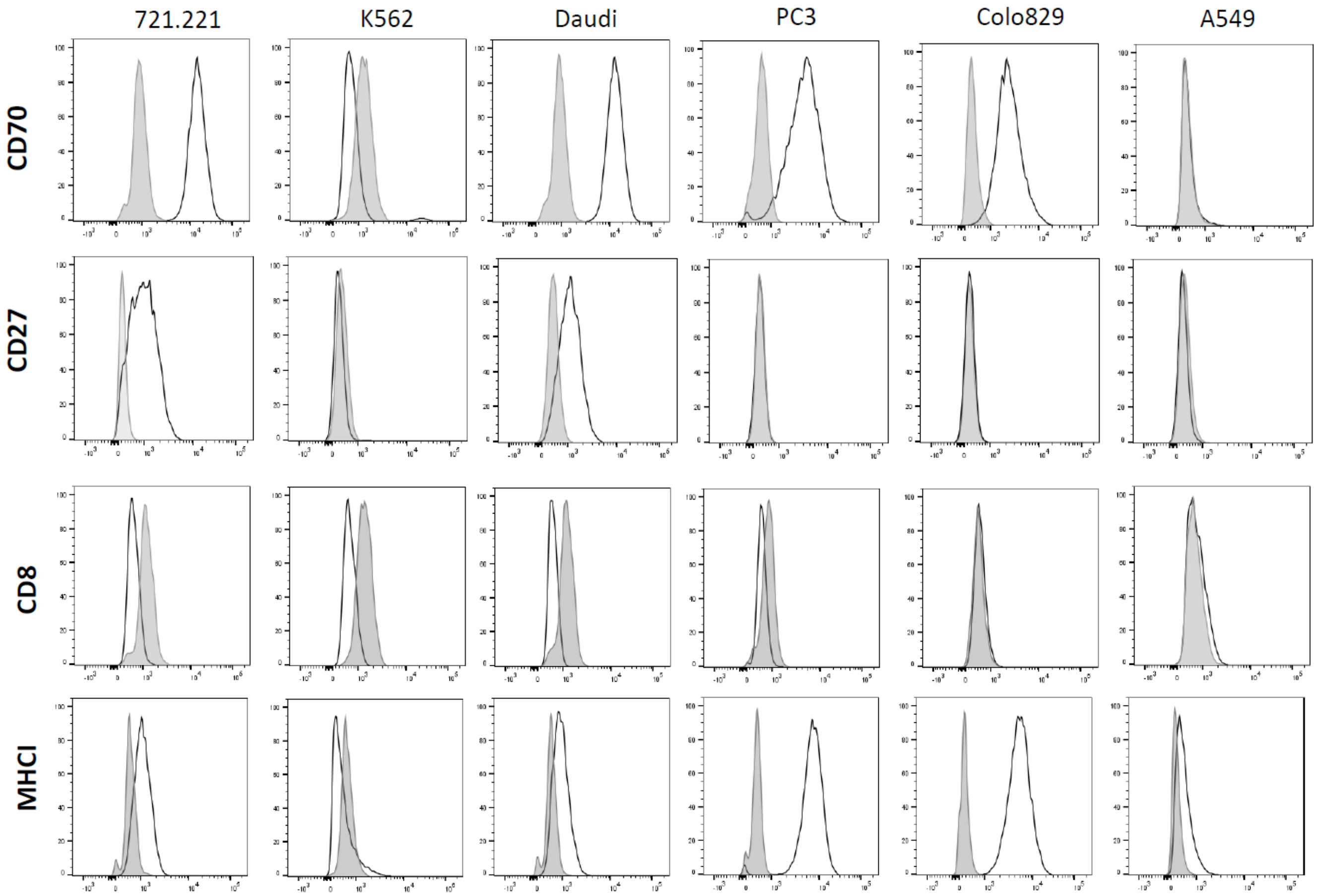

Supplementary Figure 1. Expression profiles of CD70, CD27, CD8 and MHCI molecules on 721.221, K562, Daudi, PC3, Colo829 and A549 target cancer cell lines. The target cell lines were analyzed by flow cytometry with a specific antibody (open black curve) and an isotype control (filled grey curve) coupled to the appropriate fluorophore.

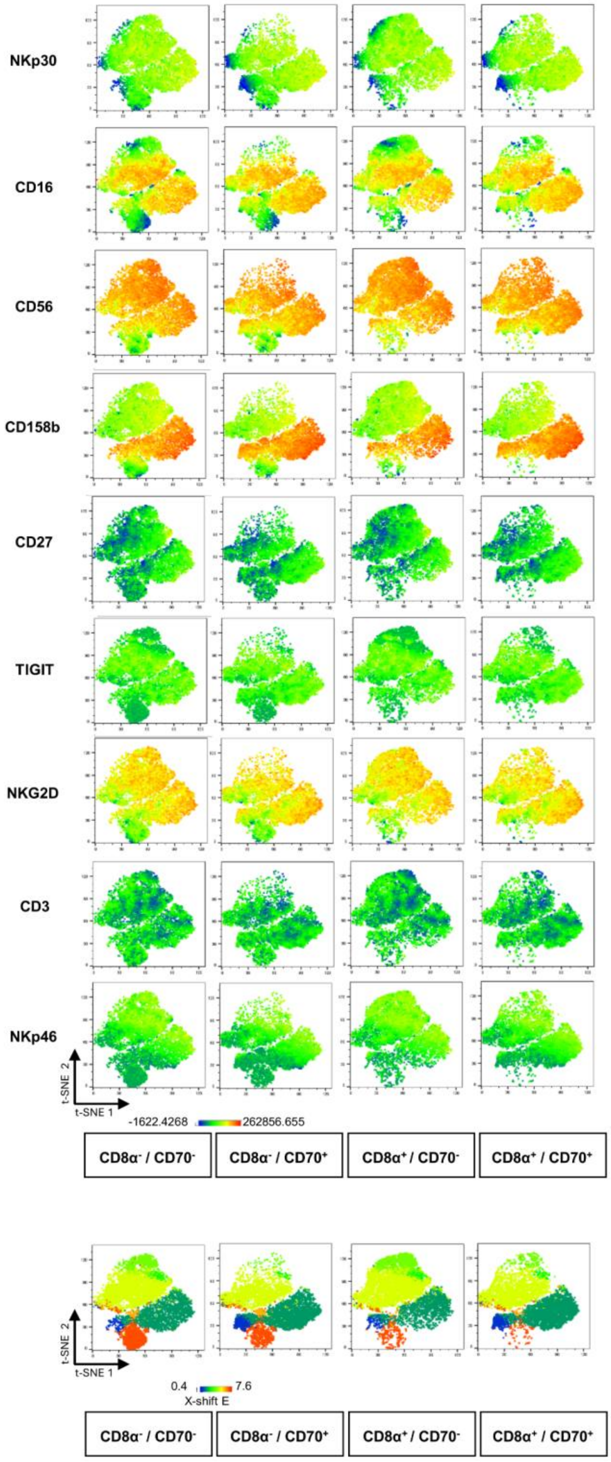

Supplementary Figure 2. Phenotypic t-SNE analysis of CD8α/CD70 subsets based on their expression of a panel of markers.
