# Supplementary material for: CD8α and CD70 mark human natural killer cell populations which differ in cytotoxicity
Source: Front Immunol. 2025 Feb 19;16:1526379. doi: 10.3389/fimmu.2025.1526379 (PMC11880019; doi:10.3389/fimmu.2025.1526379)
Supplement: Supplementary file 6 [file Table1.docx]

**Supplementary Table 1.**

Fluorescently labelled primary antibodies

| **Target** | **Fluorophore** | **Clone** | **Isotype** | **Manufacturer** | **Application** |
| --- | --- | --- | --- | --- | --- |
| **CD3** | PE | UCHT1 | IgG1_k_ | Biolegend | Cell sorting |
| **CD3** | AF700 | SK7 | IgG1_k_ | Biolegend | Clone phenotyping |
| **CD16** | BUV805 | 3G8 | IgG1_k_ | BD Biosciences | Clone phenotyping |
| **CD56** | AF488 | HCD56 | IgG1_k_ | Biolegend | Cell sorting and clone phenotyping |
| **CD56** | BV421 | HCD56 | IgG1_k_ | Biolegend | Sorting and Phenotyping panel |
| **NKG2D** | AF647 | 1D11 | IgG1_k_ | Biolegend | Phenotyping |
| **NKG2D** | APC | 1D11 | IgG1_k_ | Biolegend | Phenotyping panel |
| **NKp30** | BV785 | P30-15 | IgG1_k_ | Biolegend | Phenotyping |
| **NKp30** | BUV563 | 3G8 | IgG1_k_ | BD Biosciences | Phenotyping panel |
| **NKp46** | APC-Cy7 | 9E2 | IgG1_k_ | Biolegend | Phenotyping panel |
| **CD158b** | BV605 | DX27 | IgG2b, κ | BD Biosciences | Phenotyping panel |
| **TIGIT** | PE-Cy5 | A15153G | IgG2a, κ | Biolegend | Phenotyping panel |
| **CD8Α** | AF488 | RPA-T8 | IgG1_k_ | Biolegend | Phenotyping panel |
| **CD70** | PE | 113-16 | IgG1_k_ | Biolegend | Sorting and phenotyping panel |
| **CD27** | BV785 | O323 | IgG1k | BioLegend | Phenotyping panel |
